# Porous organic materials offer vast future opportunities

**DOI:** 10.1038/s41467-020-15911-8

**Published:** 2020-10-02

**Authors:** Tianyu Liu, Guoliang Liu

**Affiliations:** 1 Department of Chemistry, Virginia Tech, Blacksburg, VA 24061 USA; 2 Macromolecules Innovation Institute, Virginia Tech, Blacksburg, VA 24061 USA; 3 Academy of Integrated Science-Division of Nanoscience, Virginia Tech, Blacksburg, VA 24061 USA

**Keywords:** Polymers, Porous materials, Polymers

## Abstract

In light of the surging research on porous organic materials, we herein discuss the key issues of their porous structures, surface properties, and end functions. We also present an outlook on emerging opportunities, new applications, and data science-assisted materials discovery.

Porous organic materials have diverse compositions and tunable pore sizes (Fig. 1), which have enabled a wide variety of applications including separation, filtration, storage, catalysis, and drug delivery. According to the International Union of Pure and Applied Chemistry definition, the pores in the porous organic materials are classified into micropores (<2 nm), mesopores (2–50 nm), and macropores (>50 nm). It is often beneficial for porous organic materials to possess hierarchical pores across multiple length scales. The size exclusion of these pores, as well as the physical and chemical interactions of molecules with pore surfaces, offer the porous organic materials tunable permeability and selectivity that are critical to their applications.

**Fig. 1:**
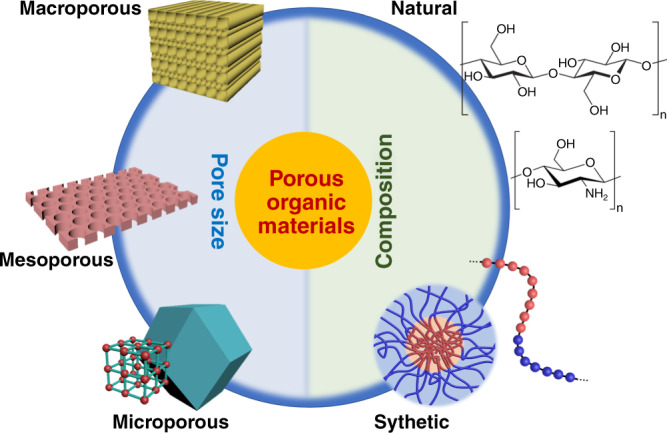
Classifications of porous organic materials based on pore size and composition. Porous organic materials can possess pores of different sizes including macropores, mesopores, and micropores. In terms of composition, the porous organic materials can be made of either natural or synthetic compounds.

## Precise control over structure, property, and function

The application of porous organic materials highly depends on their structures, properties, and functions. Controlling the structure-property-function of porous organic materials begins with the accurate synthesis of organic molecules because molecular structures dictate processing methods (including self-assembly), structures, properties, and end functions. Considering the length of this comment, we will only discuss structure and property, the two key issues ultimately determining the end functions.

The foremost structural factor is pore size. The pore size correlates with surface areas and pore volumes, and it eventually influences the end functions such as size-based permeability and selectivity for guest species. The control over macropore sizes is relatively straightforward and has been achieved by hard templating and other techniques^1^. Similarly, mesopore sizes are tunable using soft templates. Notably, the prosperous development of block copolymers over the last few decades has enabled excellent control over the size and order of mesopores at the molecular level via molecular self-assembly and directed assembly^2,3^. The control over the micropore sizes of organic polymers, however, has remained mostly an art. One exception is organic frameworks that have intrinsically narrow pore size distributions. Mechanisms have been developed to control the structures, configuration, and conformation of organic molecules, yielding some successes in controlling micropore sizes of porous polymers. Regulating the spatial arrangement of the micropores, which impacts the gas separation and filtration performances, still has vast room for improvement. For high flux and rapid mass transport, precise control over one specific pore size is often insufficient. It is necessary to prepare multiple types of pores across a range of length scales, and more importantly, to ensure their interconnectivity^4^. In addition to the pore interconnectivity, reducing structural tortuosity remains another grand challenge of porous membranes transporting guest molecules across.

Among the many properties, interfacial properties, which are primarily determined by surface functional groups, govern the wettability to liquids, binding affinity to molecules, and hence, the end function of molecular permeability and adsorption selectivity. Therefore, tuning surface functionalities is critical for optimizing the performances of porous organic materials in separation, gas storage, and biological applications. For instance, in gas separation membranes, pore surfaces decorated with functional groups can selectively interact with certain gases. One can design surface functional groups in the molecular blocks that build up the porous matrix. These functional groups become, at some point, inherent to the organic matrix. Alternatively, one can introduce surface functional groups through post-processing, that is, functionalizing the porous organic materials with moieties to modify the surface chemistries. Chemical modifications under controlled conditions of time, temperature, and pressure add functional groups to porous organic materials; however, caution must be exercised when post-treating porous organic materials with methods such as thermal annealing. Polymers have specific thermal stability and operating temperature ranges, and treating them at temperatures outside the suitable ranges will irreversibly alter their molecular structures or even degrade them. In this respect, strategies that specifically change the surface functional groups are of paramount importance for accurate tunability. Notably, functional groups of tunable compositions, densities, and spatial distributions can open new applications of porous organic materials. This goal is potentially achievable by borrowing strategies from other communities. For example, to control the composition of surface functionalities of graphene electrocatalysts, spatial confinement directs the formation of planar N-dopants (pyridinic- and pyrrolic-nitrogen) over non-planar quaternary N-dopants^5^.

## Emerging opportunities and applications

Thanks to precise control over the structure, property, and function, porous organic materials have found great use in separation, filtration, and storage, and the applications are poised to expand in the ensuing decades. Although porous organic materials are versatile, their applicability under extreme conditions is still limited, for example, under extremely high or low temperatures, extremely high tension, and exposure to high-energy radiation that are associated with space exploration. Developing new porous organic materials, especially those with uncompromised thermal stability, mechanical strength, and processability, challenges future research on porous organic materials. Exploiting existing high-performance engineering polymers, such as polyimide, in a porous form, represents a straightforward yet effective strategy^6,7^. The innovation of chemistry to synthesize high-performance porous polymers will be an enormous opportunity for polymer chemists. Furthermore, from a processing perspective, ensuring controllability over the porous structures is required and can be challenging.

One breakthrough in porous organic materials is porous organic frameworks, including covalent organic frameworks (COFs) and metal-organic frameworks (MOFs)^8^. Both COFs and MOFs have distinct organic building blocks. Recently their organic building blocks have embraced novel properties. For example, redox-active benzoquinone-based COFs^9^ have revolutionized the conventional notions that porous organic materials are poor electrical conductors and inert for electrochemical energy storage. These conductive porous materials provide both high capacities and rate capability, which are often mutually exclusive for conventional electrode materials. The success will motivate future investigations and expand the applications of porous organic materials. One can envision future organic porous materials as separators for safe and long-lasting batteries, catalyst supports for extraordinary reactant diffusion and product selectivity, and gas separation membranes with outstanding selectivity and permeability.

Highly controlled structures enable systematic mechanistic investigations. Porous organic materials with uniform pore sizes or distinct functionalities minimize the number of variables and simplify the models for computational simulations. For example, in capacitive energy storage, molecular dynamics can simulate the interplays among pore widths, capacitances (or capacities), and rate capabilities^10^. Unfortunately, the lack of porous organic materials with uniform pores and homogeneous functional groups hinders experimental verifications of the simulations. Electrically conductive porous organic frameworks, with their highly uniform pore sizes and functionalities, are emerging electrochemical materials that can potentially enable such mechanistic studies. Pioneering work on charge-storage mechanisms of COFs^9^ and MOFs^11^ are recent examples. High-carbon-yield porous organic materials can function as templates to prepare porous carbon powders^12^, films^13^, and fibers^14^. Notably, porous carbon fibers from porous organic materials have shown outstanding electrical and ion conductivities^14^, as well as capability of hosting guest materials and allowing for fast charging and discharging^4^.

## Emerging materials development by computation and data science

Computational tools such as molecular simulations have always been indispensable to propel the development of porous organic materials. Emerging methods such as machine learning and data science are powerful to accelerate the discovery, design, synthesis, processing, and evaluation of new porous organic materials. The research of porous organic materials has accumulated countless data and information in the literature, but most of them are intuitional or empirical. Manually extracting, sorting, and analyzing the massive data become increasingly challenging. Machine learning and data science, however, can help collect and analyze enormous combinations of materials metrics, including compositions, morphologies, pore sizes/volumes, and surface chemistries. They can assist the identifications of porous organic materials with the most desirable characteristics. They can aid experiments in screening possible synthetic routes and suggesting the most affordable and efficient means. For example, inverse design, a technique predicting compositions and structures of starting materials based on end-of-use functions^15^, can accelerate the discovery of novel porous organic materials.

**Fig. 2:**
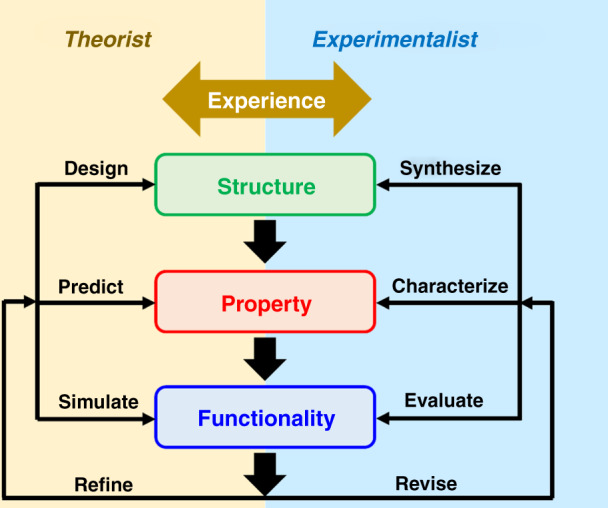
Scheme illustrating the potential collaborations between theorists and experimentalists. Theorists design the structures, predict the properties, and simulate the functionalities of porous organic materials, which can be refined with input from experimentalists. Exprimentalists synthesize molecules with tailored structures, characterize the properties, and evaluate the functionalities of porous organic materials, with the input from theorists to revise the experimental protocols. The efficient information and experience exchanges between theorists and experimentalists will expedite the development of porous organic materials.

The advancement of porous organic materials will benefit from interdisciplinary efforts. Joint endeavors by theorists and experimentalists from both science and engineering fields will accelerate the identification of preferable structures, properties, and functionalities for targeted applications (Fig. 2). Extensive and frequent exchanges of ideas and knowledge are most preferred for effective cooperation. The alliance of researchers with diverse backgrounds and specialties are mutually beneficial: Theorists provide insightful guidelines to reduce the burdens of materials synthesis and structure optimization, while experimentalists return with first-hand experimental data for model refinement; Scientists offer fundamental knowledge for materials synthesis and processing, whereas engineers repay with mass production and performance evaluation of newly developed porous organic materials.

## References

[1] Wu D, Xu F, Sun B, Fu R, He H, Matyjaszewski K (2012). Design and preparation of porous polymers. Chem. Rev..

[2] Serrano JM (2019). Composition design of block copolymers for porous carbon fibers. Chem. Mater..

[3] Seo M, Hillmyer MA (2012). Reticulated nanoporous polymers by controlled polymerization-induced microphase separation. Science.

[4] Liu T, Zhou Z, Guo Y, Guo D, Liu G (2019). Block copolymer derived uniform mesopores enable ultrafast electron and ion transport at high mass loadings. Nat. Commun..

[5] Ding W (2013). Space-confinement-induced synthesis of pyridinic- and pyrrolic-nitrogen-doped graphene for the catalysis of oxygen reduction. Angew. Chem. Int. Ed..

[6] Guo D, Khan AU, Liu TY, Zhou ZP, Liu GL (2019). Sub-10 nm domains in high-performance polyetherimides. Polym. Chem..

[7] Xu Z, Liu T, Cao K, Guo D, Serrano JM, Liu G (2020). Thermally stable and mechanically strong mesoporous films of polyetherimide-based triblock copolymers. ACS Appl. Polym. Mater.

[8] Yaghi OM (2019). Reticular chemistry in all dimensions. ACS Cent. Sci.

[9] Shi R (2020). Nitrogen-rich covalent organic frameworks with multiple carbonyls for high-performance sodium batteries. Nat. Commun..

[10] Zhan C (2017). Computational insights into materials and interfaces for capacitive energy storage. Adv. Sci..

[11] Bi S (2020). Molecular understanding of charge storage and charging dynamics in supercapacitors with MOF electrodes and ionic liquid electrolytes. Nat. Mater..

[12] Zhong M (2012). Electrochemically active nitrogen-enriched nanocarbons with well-defined morphology synthesized by pyrolysis of self-assembled block copolymer. J. Am. Chem. Soc..

[13] Zhou Z, Liu G (2017). Controlling the pore size of mesoporous carbon thin films through thermal and solvent annealing. Small.

[14] Zhou Z, Liu T, Khan AU, Liu G (2019). Block copolymer-based porous carbon fibers. Sci. Adv..

[15] Kim B, Lee S, Kim J (2020). Inverse design of porous materials using artificial neural networks. Sci. Adv..

